# Vesical Haemangioma in Klippel-Trenaunay-Weber Syndrome: A Clinical Case Report

**DOI:** 10.7759/cureus.49952

**Published:** 2023-12-05

**Authors:** Ahmed Mohamed, Yew Fung Chin, Mohamed Farah, Wasim Mahmalji

**Affiliations:** 1 Urology, Wye Valley NHS Trust, Hereford, GBR; 2 General Surgery, Royal Shrewsbury Hospital, Shrewsbury, GBR

**Keywords:** urinary tract involvement, multidisciplinary approach, laser fulguration, vesical haemangioma, klippel-trenaunay-weber syndrome

## Abstract

Klippel-Trenaunay-Weber (KTW) syndrome, a rare vascular disorder, often presents with cutaneous capillary malformations and soft tissue hypertrophy. However, urinary tract involvement in the form of vesical haemangiomas is a seldom-encountered clinical condition. We present a case of a 37-year-old male with KTW syndrome who exhibited recurrent gross haematuria, prompting clinical evaluation. Initial diagnostic assessments revealed erythematous changes in the bladder, consistent with haemangiomas. Despite an initial biopsy and diathermy, the patient's symptoms recurred, leading to a subsequent management strategy involving laser fulguration. This case underscores the significance of recognizing cutaneous haemangiomas as potential indicators of urinary tract involvement in KTW syndrome and highlights the challenges in managing vesical haemangiomas, where a multidisciplinary approach is essential for optimal care.

## Introduction

Klippel-Trenaunay-Weber (KTW) syndrome is a rare vascular disorder encountered in 1 in every 30000 live births [1,2]. It was described by Klippel and Trenaunay in 1900 and was further described by Parkes Weber in 1907 [2,3]. The syndrome is characterized by diverse clinical features, including cutaneous capillary malformations, varicose veins, soft tissue, and bone hypertrophy [3-5]. While KTW typically presents with prominent cutaneous manifestations, its involvement in the urinary tract, particularly through the development of bladder haemangiomas, represents a seldom-encountered clinical condition [5,6].

In KTW syndrome with urinary tract involvement, patients often present with gross haematuria, and the diagnostic utility of cystoscopy in identifying bladder haemangioma involvement is well established [6]. However, managing such cases can be challenging. Historically, partial cystectomy has been the standard of care for large, life-threatening haemangiomas, although less invasive approaches have been proposed [6,7].

To address the management hurdles associated with KTW syndrome affecting the urinary tract, the current case report could provide valuable insights, contributing to our understanding of this rare and often complex condition.

## Case presentation

A 37-year-old male presented to the urology clinic due to recurrent episodes of visible haematuria accompanied by intermittent suprapubic cramping and increased urinary frequency over around 18 months. The patient reported no history of trauma or other lower urinary tract symptoms (LUTS). On systematic review, he denied any symptoms of anemia, weight loss, loss of appetite, or cardiopulmonary symptoms. Besides KTW and congenital hemi-hypertrophy, his past medical and surgical history was unremarkable. The only notable aspect of the patient's social history was a history of smoking equivalent to 2.5 pack years. The clinical examination revealed no clinically appreciated abnormalities observed in the abdomen or groin.

Further investigations were carried out to identify the cause of haematuria. A computed tomography urography (CTU) was conducted, which revealed no abnormalities in the urinary tract; however, multiple haemangiomas were detected in the spleen, liver, and right adrenal gland. The flexible cystoscopy revealed global erythematous changes in the bladder, with a few dark areas near the dome of the bladder, likely representing a haemangioma. Notably, no abnormalities were observed during the cystoscopy examination of the urethra and prostate.

Following this, a subsequent cystoscopy was conducted under general anesthesia, during which two biopsies were procured from the bladder, including the suspected haemangioma, which was subsequently subjected to diathermy. Following this procedure, the patient was reassured and discharged back to their general practitioner (GP).

**Figure 1:**
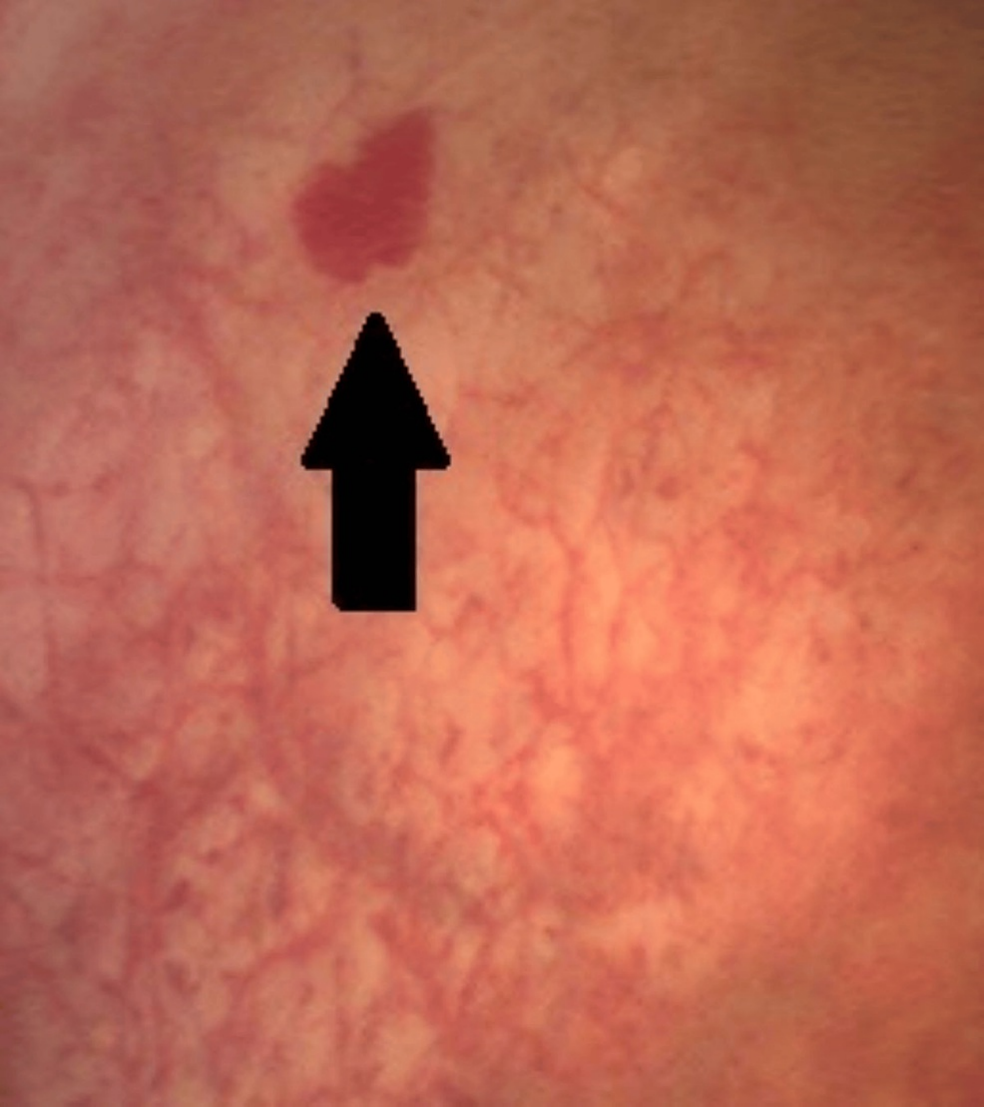
Flexible cystoscopy findings Flexible Cystoscopy showing a small Vesical haemangioma located near to the dome of the bladder (black arrow).

The patient was re-referred to urology six years after being initially discharged for recurring visible haematuria. Subsequent assessment with flexible cystoscopy identified the diathermy site and prior biopsy on the left lateral wall of the bladder, together with evident haemangiomas and widespread erythematous alterations in the bladder (Figure 1). No anomalies in the urinary system were detected on the CT scan. Following a multidisciplinary discussion, the best intervention will be laser fulguration of the bladder haemangiomas using a Holmium-YAG laser. In this case, the laser configuration utilized a power output of 1 joule operating at a frequency of 10 Hz.

## Discussion

The presented clinical case is aligned with historical observations and a contemporary understanding of the rarity of KTW. The presence of cutaneous haemangiomas, often a defining feature of KTW syndrome, serves as a crucial clinical clue alerting urologists to the potential existence of urinary tract haemangiomas, particularly when patients present with recurrent and painless gross haematuria similar to our case.

Managing vesical haemangiomas in individuals with KTW syndrome can pose a considerable challenge. In asymptomatic patients with KTW syndrome, a conservative approach is typically recommended [1,5,8]. Historically, endoscopic resection was considered but later discouraged due to perceived hazards related to its inability to effectively seal large, deep vessels within the muscular layer that are encountered during the procedure [1,2,9]. As a result of this, endoscopic resection is presently not indicated for such haemangiomas.

In cases where less invasive treatments prove unsuccessful, partial cystectomy becomes a viable option, considering the substantial morbidity associated with this surgical intervention, including reduced bladder storage function and impaired voiding capabilities [1,5,8]. Notwithstanding these risks, partial cystectomy may be warranted, particularly in cases of life-threatening haematuria.

While various less invasive treatment alternatives have been proposed, each comes with its own specific limitations and associated risks of complications. The use of systemic interferon-alpha-2 therapy was proposed; however, the available evidence is currently insufficient to establish its efficacy conclusively [2]. Selective embolization of the internal iliac arteries is also a proposed approach, but it is not the preferred option due to the potential for rapid collateralization [1]. Another therapeutic option is radiotherapy; however, some case reports indicate that the morbidity can be severe and may cause complications such as a contracted bladder, with only short-term positive outcomes demonstrated (5).

Endoscopic laser fulguration has shown promise in early studies, offering an attractive option due to its minimally invasive nature and the widespread availability of laser technology in contemporary urology practice [1,5,9,10]. Furthermore, the advantageous factor of a small-size bladder haemangioma, as observed in our case, amplifies the favourability of this technique for treating vesical haemangioma in our patient. Nevertheless, the choice of management should be tailored to the specific characteristics of each case, and a multidisciplinary approach involving radiologists, urologists, and vascular surgeons is strongly recommended to ensure comprehensive and effective care.

Furthermore, the characteristic locations of bladder haemangiomas in KTW syndrome, predominantly on the anterior bladder wall and dome, offer insights into the nature of these lesions. Rare trigone and bladder neck involvement further characterize their distinctive distribution [2,9].

The presented case is emblematic of the recurrent and painless gross haematuria that frequently serves as the initial clinical presentation of bladder involvement in KTW syndrome. The etiological underpinnings of these lesions remain speculative, with theories suggesting venous overload as a possible mechanism [3,6,7].

## Conclusions

This clinical case aligns with the broader clinical understanding of KTW syndrome and its associated bladder haemangiomas. The importance of recognizing the clinical significance of cutaneous haemangiomas as indicators of potential urinary tract involvement cannot be overstated. When managing such cases, a tailored and multidisciplinary approach is imperative to effectively address the intricate nature of KTW syndrome and its complications.
